# Acculturative Stress and Influential Factors among International Students in China: A Structural Dynamic Perspective

**DOI:** 10.1371/journal.pone.0096322

**Published:** 2014-04-30

**Authors:** Bin Yu, Xinguang Chen, Shiyue Li, Yang Liu, Angela J. Jacques-Tiura, Hong Yan

**Affiliations:** 1 School of Public Health, Wuhan University, Wuhan, Hubei, P. R. China; 2 Global Health Institute, Wuhan University, Wuhan, Hubei, P. R. China; 3 Pediatric Prevention Research Center, Wayne State University, Detroit, Michigan, United States of America; 4 Department of Epidemiology, University of Florida, Gainesville, Florida, United States of America; City University of Hong Kong, Hong Kong

## Abstract

Stress represents a prominent aspect of modern life and is associated with numerous negative health consequences. International students are a key force in shaping globalization. However, these students often experience acculturative stress, influencing their health and well-being. The growing number of international students in China emerges as a new global health challenge and presents an opportunity to advance our understanding of acculturative stress. This study aims to investigate the acculturative stress of international students in China, and verify the mechanism and influential factors of acculturative stress. We analyzed survey data from 567 international students attending universities in Wuhan, China. We used a network-based analytical approach to assess the structure of the Acculturative Stress Scale for International Students and used regression analysis to assess the relationships between acculturative stress and theoretically related factors. We found that higher levels of acculturative stress were reported by students from Asia and Africa than from other regions (Europe/America/Oceania). Lower acculturative stress was reported by unmarried students than others and by students well prepared than not well prepared. We verified seven acculturative stress subconstructs: rejection, identity threat, opportunity deprivation, self-confidence, value conflict, cultural competence, and homesickness; and discovered a three-dimensional network structure of these subconstructs. Our results suggest that acculturative stress was more common among international students in China than in developed countries. Acculturative stress was also more common among international students who did not well prepared, married, and belonged to an organized religion. African and Asian students' stress was higher than that for students from other regions. Acculturative stress prevention programs should seek to improve preparedness of the international students for studying abroad and pay extra attention to the high risk subgroups.

## Introduction

Stress represents a prominent aspect of modern life [1]. Both acute and chronic stressors have been associated with a number of negative health consequences, including ischemic heart disease, hypertension, cancers, diabetes, neurodegenerative diseases, depression, substance use, eating disorders, suicide, and other health problems [2]. Stress exposure may cause heart dysfunction by altering the balance between the vagal and sympathetic systems [3]. Stress can also cause protein misfolding in brain cells, activating a cascade of deleterious molecular events that disturbs normal cell functioning, leading to emotional and neurological diseases [4].

Acculturative stress – the process of confronting challenges in cross-cultural exchange settings – is a specific form of stress that represents a growing threat to the health of many populations, particularly international students, as the pace of globalization continues to accelerate [5], [6]. While studying to develop themselves, international students also serve as living bridges for exchanging knowledge, techniques, culture, and lifestyles across the globe [7]. In addition to contributing to economic globalization, these pioneers play a leading role in promoting global health. At the same time, researches indicate that due to difficulties in adapting to a new social and cultural environment (i.e., acculturative stress), stress-related physical, psychosocial, and behavioral problems are prevalent among international students [5], [7].

Acculturative stress has traditionally been investigated among various mobile populations in developed countries [8], [9]. Along with the rapid economic development, China's international student population has risen dramatically in the past two decades. China is now hosting approximately 328,000 students from more than 200 countries and regions across the globe [10]. While most international students studying in developed countries are from developing countries; the international student population in China includes those from both developed and developing countries [10]. The Chinese socioeconomic and cultural settings, the diverse origins and the growing number of international students in China create a window of opportunity to investigate acculturative stress in a novel region.

Much progress has been made in understanding acculturative stress. With data collected in different settings, researchers have identified a number of components of acculturative stress. The following seven components are of great significance for stress reduction: (1) homesickness [11], (2) rejection [12], (3) opportunity deprivation [12], (4) identity threat [13], (5) cultural competence [14], (6) value conflict [15], and (7) self-confidence [16]. However, few studies have investigated potential mechanisms linking these components to form a structural understanding of acculturative stress.

International students frequently jointly experience rejection, identity threat and opportunity deprivation [7], [17]; researchers have shown these three constructs to be highly correlated with each other [17], [18]. Together, they form *assaultive socio-environmental barriers* to smooth acculturation, leading to stress. Another three highly correlated components, adequate competence for cross-culture communication, avoidance of value conflict, and adequate self-confidence, form a *protective fortress* against stress [12], [16], [17]. International students often report homesickness [7]; however, acculturation theory suggests that homelessness may become stressful in and of itself only after protective coping mechanisms fail [12]. Our primary goal in this study is to test the dynamic structure of the seven components of acculturative stress.

Researchers have documented several potential risk and protective factors of acculturative stress. Study findings from international student samples in developed countries suggest that lack of preparedness [19], shorter length of stay [20], lower language competency [21], greater cultural dissimilarity [7], lower educational attainment [22], and lower income [7] were important influential factors. Age [23], religion [24], and marital status [7] have been inconsistently related to acculturative stress. We do not know whether and to what extent any of these factors are also related to acculturative stress among international students in China.

In this study, we addressed the following four aims: (1) To document acculturative stress among international students in China with the published Acculturative Stress Scale for International Students (ASSIS); (2) to empirically verify the seven subconstructs derived from ASSIS; (3) to investigate potential underlying dynamics of acculturative stress by relating the seven subconstructs with a network-based analytical approach; and (4) to examine both protective and risk factors associated with acculturative stress, including the overall level and the individual subconstructs.

## Materials and Methods

### Ethics Statement

The survey protocol was approved by the Institutional Review Board at Wuhan University, China, and the survey data analysis was approved by the Human Investigation Committee at Wayne State University, USA.

### Participants and Sampling

For this study, we recruited international students who were attending colleges and universities in Wuhan, China without Chinese citizenship. Wuhan, the provincial capital of Hubei Province, is located in central China by the Yangtze River with a total population of more than nine million and per capita GDP of $10,335 in 2011. Wuhan is also known as a university city, hosting 79 colleges and universities. Government statistics indicate that 11,932 international students from 161 countries were enrolled in colleges and universities in Wuhan in 2012. Participants were selected from the four largest comprehensive universities, which accounted for 59% of the total international student population in Wuhan.

All international students in the participating schools were eligible to be included in the study. School administrators provided access to international students' classes. Students were recruited by class, the smallest organized unit for schooling in China. Different from international students in many western countries, the majority of international students in China take classes together with only their fellow international students. Recruitment and survey administration were carried out in a classroom immediately after students completed a class and did not have another class thereafter. Before survey administration, students were fully informed about the study.

All eligible students were encouraged to participate and data were collected only among those who agreed to participate by signing the informed consent. All students we approached agreed to participate and no one withdrew from the study early.

### Data Collection and Processing

Data were collected using the International Student Health and Behavior Survey (ISHBS). We developed the instrument with previously validated measures. The ISHBS covers of demographic and general information, experiences of working and daily life, cultural adaption, acculturative stress, and other variables. Most participants completed the full survey in 30–45 minutes. Pilot testing with 51 international students indicated that the survey was easily understood and well accepted. Participants were offered the opportunity to complete the questionnaire in either English or Chinese. Preliminary analyses indicated no significant differences in the data collected with the Chinese version (n = 62) and the English version (n = 505) of the questionnaires for any variables used in this study.

The survey was conducted in the spring semester in 2012. Three trained senior-year graduate students recruited participants and distributed the questionnaires. To ensure confidentiality and privacy, participants sat in a classroom with at least one empty seat between any two students. The survey was anonymous. Participants were allowed to skip any questions they did not want to answer. After they completed the survey, participants sealed their questionnaire in an envelope we provided and dropped it into a locked box.

The data manually were entered into the computer with EpiData software by the data collectors. We used a double-entry protocol to minimize data-entry errors. Among the 630 completed questionnaires, 63 were excluded because of missing data on key variables, yielding a final sample of 567 (90%) participants.

### Measures

#### Acculturative Stress

Acculturative stress was measured with the 36-item Acculturative Stress Scale for International Students (ASSIS) [12]. Several studies have reported Cronbach's α>.90 for the ASSIS [12], [21]. Participants indicated their level of agreement on a 1, *strongly disagree*, to 5, *strongly agree*, scale. Cronbach's α of our data was .93. Scale scores were computed by summing up item scores; larger scores indicated higher levels of acculturative stress.

#### Acculturative Stress Subconstructs

To further our understanding of acculturative stress, we identified seven independent but also logically connected subconstructs among the ASSIS items. We established the subconstructs through a team effort. We first proposed a number of constructs directly related to acculturative stress through intensive literature review, brainstorming, and group discussions. With a set of tentative subconstruct names, each researcher attempted to match individual ASSIS items with the proposed constructs independently, followed by group discussions to resolve discrepancies. The tentatively matching results were then subjected to intensive psychometric evaluation to fine-tune item-subconstruct match such that (1) the number of items per subconstruct were similar, (2) the Cronbach's α coefficient was maximized, and (3) data-model fit was optimized.

#### Key Influential Factors

Country/place of origin was assessed using the question: “What is your nationality?” Participants by country were grouped into Africa, Asia, and others (Europe, Americas, and Oceania) after an assessment of the frequencies across countries. Religious beliefs (five categories: none, Buddhist/Hindu, Christian, Muslim, and others) were assessed using a checklist. Length of stay in China was assessed using the question: “How long have you been staying in China?” (number of years and number of months). Level of preparedness for studying in China was assessed with, “How could you rate your preparations for coming to China to study?” rated on a 1, *not at all well prepared*, to 4, *very well prepared*, scale. We then collapsed responses into *not well prepared* (from *not at all well* and *somewhat not well*) and *well prepared* (from *somewhat well* and *very well*). Demographic factors were age (in years), gender (male or female), marital status (unmarried or others, including married, divorced, separated and other statuses), educational attainment before coming to China (undergraduate and graduate), and current major (four categories: science, literature/business/law, medicine, and others).

### Statistical analysis

Systematic psychometric assessment was used to assess the ASSIS as a whole and its seven subconstructs. We evaluated item responses, internal consistency and reliability (Cronbach's α), and one- and two-level CFA (measurement modeling). For reliability analyses, Cronbach's α≥.7 was set as acceptable, .8 as good; and .9 as great. For measurement modeling analyses, data-model fitting were assessed using the following four indices (and benchmarks): GFI (>.9), CFI (>.9), RMSEA (<.05) and Chi-square/df (<2) [25].

A network analysis approach [26] was applied to explore the relationship among the seven acculturative stress subconstructs. In this approach, we first computed the correlation coefficients for all pairs of the seven constructs. We then used a network graph to represent the relationship for any pairs with a moderate or higher level of correlation (*r*≥.40).

In assessing factors associated with acculturative stress and its seven subconstructs, Student t-tests (two groups) and ANOVA (multi-groups) were used first. Significant results from the bivariate analysis (*p*<.1) were further verified with multiple regression models to control demographic and other covariates. Type I error was set at *p*<.05 (two-sided). All analyses were conducted using SAS version 9.3 (SAS Institute, Cary, NC).

## Results

### Sample characteristics

The 567 participants came from 94 countries and regions across the globe. Participant characteristics are presented in Table 1. Nearly 60% of participants were men, 84% of the sample came from Africa or Asia, mean age was 23 years, and median age was 22 years. Just under one-quarter of the sample had prior graduate work before studying in China, almost 90% of the sample was unmarried, and Christian and Muslim were the most commonly reported religious beliefs. Most students were studying literature, business, law, or medicine. On average, students had been in China just over one year, and approximately three-quarters indicated feeling well prepared for studying in China.

**Table 1:** Selected characteristics of the study sample.

Characteristic	Men	Women	Total
**Total, n (%)**	336 (59.26)	231 (40.74)	567 (100.00)
**Place of origin, n (%)**			
Africa	147 (44.82)	76 (33.93)	223 (40.40)
Asia	132 (40.24)	110 (49.11)	242 (43.84)
Others (Europe/Americas/Oceania)	49 (14.94)	38 (16.96)	87 (15.76)
**Age (in years)**			
Range	18–40	18–38	18–40
Mean (SD)	23.36 (4.43)	21.85 (3.40)	22.75 (4.11)
Median (IQR)	22.00 (5.00)	21.00 (3.00)	22.00 (4.00)
**Previous education, n (%)**			
Undergraduate	240 (74.30)	179 (79.20)	419 (76.32)
Graduate	83 (25.70)	47 (20.80)	130 (23.68)
**Marital status, n (%)**			
Unmarried	291 (87.65)	210 (91.70)	501 (89.30)
Others	41 (12.35)	19 (8.30)	60 (10.70)
**Religion, n (%)**			
None	42 (12.54)	27 (11.89)	69 (12.28)
Christian	113 (33.73)	88 (38.77)	201 (35.77)
Muslim	133 (39.70)	52 (22.91)	185 (32.91)
Buddhist/Hindu	43 (12.84)	58 (25.55)	101 (17.97)
Others	4 (1.19)	2 (0.88)	6 (1.07)
**Major area, n (%)**			
Science	57 (17.27)	21 (9.21)	78 (13.98)
Literature/business/law	116 (35.15)	79 (34.64)	195 (34.95)
Medicine	101 (30.61)	96 (42.11)	197 (35.30)
Other	56 (16.97)	32 (14.04)	88 (15.77)
**Months in China**			
Range	1–59	0–58	0–59
Mean (SD)	14.77 (12.28)	13.98 (11.88)	14.45 (12.11)
Median (IQR)	9.00 (10.00)	9.00 (8.00)	9.00 (10.00)
**Preparedness, n (%)**			
Not well prepared	73 (23.70)	58 (26.61)	131 (24.90)
Well prepared	235 (76.30)	160 (73.39)	395(75.10)

**Note**: IQR: Inter-quarter range.

### Acculturative stress and subconstructs

Table 2 indicates that the ASSIS overall was highly reliable and CFA analysis indicated that the model fit the data well (GFI = .90, CFI = .93, RMSEA = .04, and Chi-square/df = 1.89). The seven ASSIS subconstructs were: *identity threat* (IT), *opportunity deprivation* (OD), *rejection* (RJ), *self-confidence* (SC), *cultural competence* (CC), *value conflict* (VC), and *homesickness* (HS). Results from EFA indicated that these seven ASSIS subconstructs each is unidimensional and results from CFA indicated that the data fit a two-level measurement model very well (CFI = .93, GFI = .91, RMSEA = .04, and Chi-square/df = 1.90).

**Table 2:** Psychometric Characteristics of the Acculturative Stress Inventory for International Students Total Score and Seven Subconstructs.

ASSIS and subconstructs	Mean (SD)	r, item-total	α	CFI	GFI
**ASSIS Total**	92.81 (23.93)	1.00	0.93	0.93	0.90
**Rejection (RJ)**	2.34 (0.84)	0.84	0.79	0.99	0.99
**Identity threat (IT)**	2.56 (0.77)	0.85	0.64	1.00	0.99
**Opportunity deprivation (OD)**	2.58 (0.87)	0.80	0.70	0.88	0.96
**Value conflict (VC)**	2.63 (0.89)	0.80	0.67	0.99	0.99
**Self-confidence (SC)**	2.45 (0.82)	0.84	0.66	0.95	0.98
**Cultural competence (CC)**	2.54 (0.77)	0.74	0.61	0.96	0.99
**Homesick (HS)**	2.98 (0.88)	0.69	0.66	0.89	0.97

**Note**: Results presented in this table were from the one-level measurement modeling analysis for the overall ASSIS and the seven subconstructs individually. A two-level model was also conducted to assess the seven subconstructs integratively with excellent data-model fit (GFI = 0.91, CFI = 0.93, RMSEA = 0.04, and Chi-square/df = 1.90; results not shown in the table).

### Internal relationships among the seven acculturative subconstructs

**Figure 1:**
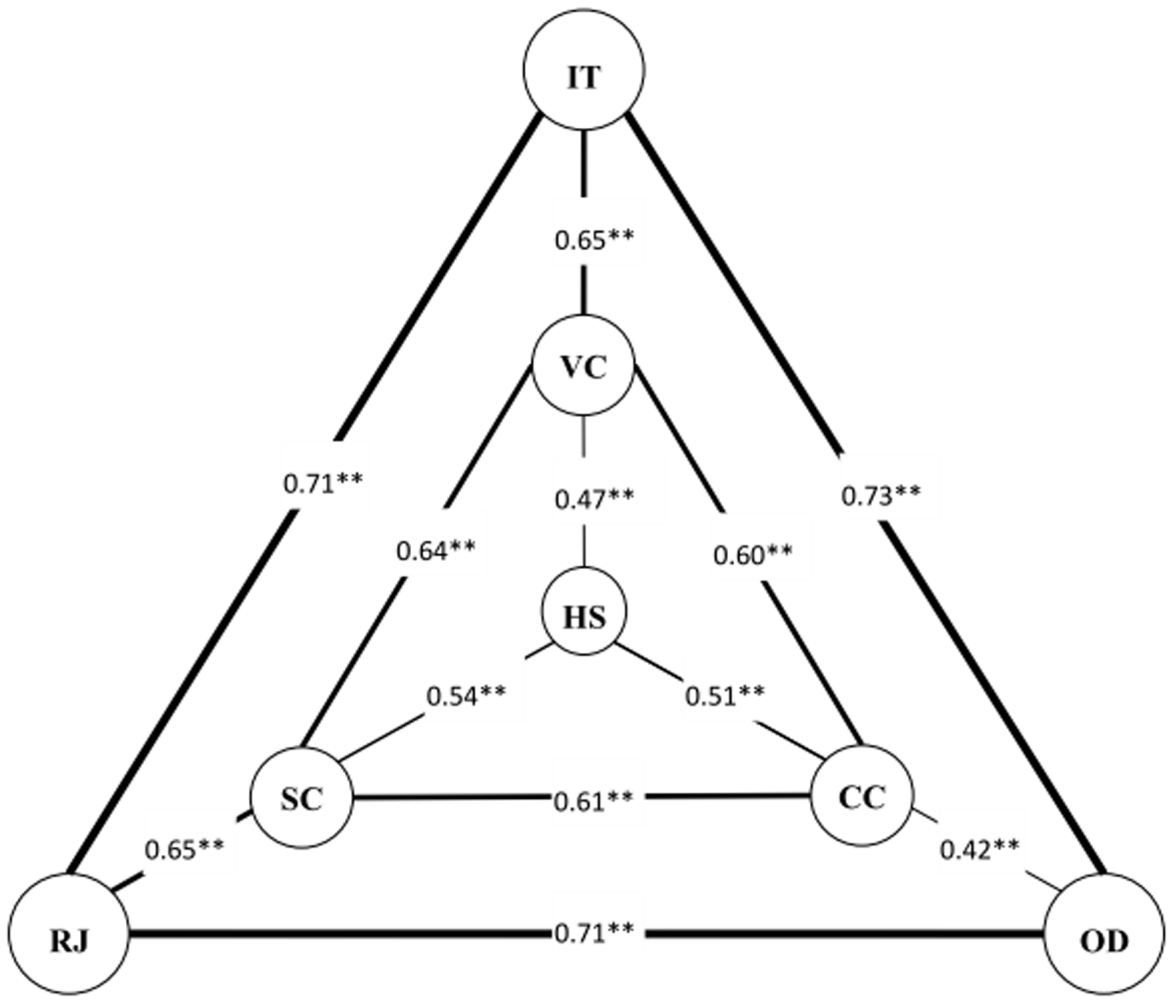
3D Structure of the Seven Acculturative Stress Subconstructs: Acculturative Stress Scale for International Students. Notes: RJ =  Perceived Rejection, OD =  Opportunity deprivation, IT =  Identity threat, SC =  Self-confidence, CC =  Cultural competence, VC =  Value conflict, HS =  Homesickness. The correlation coefficient between RJ and HS, between OD and HS and between IT and HS was 0.43, 0.49 and 0.38 respectively (not shown in the figure). Note: **p<0.001.

Figure 1 displays the results of the network analysis. The thickness of a line linking two subconstructs is proportionate to the correlation coefficient's magnitude. The seven subconstructs formed a *3D triangle* structure. The correlations among the first group of subconstructs (IT, OD, and RJ) at the outside triangle were the highest (*r*'s>.70); followed by the second group of three subconstructs (VC, CC, and SC) (.60<*r*<.70). Correlations among the remaining subconstructs varied from .42 to .65. In addition, the correlations were high between IT and VC, and between RJ and SC.

### Factors associated with acculturative stress

Data in Table 3 indicate that compared to students from other countries in Europe, America and Oceania, students from Africa and Asia scored significantly higher on ASSIS. Unmarried students reported significantly lower ASSIS scores than other students. Students who were not well prepared for studying in China scored significantly higher than well-prepared students.

**Table 3:** Relationships between Acculturative Stress and Influential Factors.

Variables	N	Total ASSIS scores	Statistic F/t (df)	P value
**Gender**			0.31 (534)	0.76
Male	316	93.07 (24.45)		
Female	220	92.43 (23.21)		
**Age in years**			0.50 (535)	0.68
≤20	190	94.31 (22.23)		
21–25	253	91.52 (24.77)		
26–30	66	93.29 (25.33)		
30+	27	93.15 (24.57)		
**Educational attainment**			0.62 (518)	0.53
Undergraduate	400	93.14 (23.52)		
Graduate	120	91.59 (25.04)		
**Place of origin**			14.91 (520)	**0.00**
Africa	208	97.66 (23.16)		
Asia	229	92.54 (22.03)		
Others (Europe/Americas/Oceania)	84	81.19 (27.03)		
**Religion**			2.09 (530)	0.08
None	64	85.00 (24.17)		
Buddhist/Hindu	99	92.90 (22.51)		
Christian	193	94.33 (24.44)		
Muslim	170	94.05 (23.41)		
Others	5	87.80 (32.76)		
**Marital status**			−2.20 (528)	**0.03**
Unmarried	475	91.99 (23.45)		
Others	55	99.45 (26.66)		
**Preparedness**			2.99 (499)	**0.003**
Not well prepared	124	97.48 (23.84)		
Well prepared	377	90.20 (23.47)		
**Major area**			0.35 (527)	0.79
Science	69	91.48 (23.54)		
Literature/business/law	187	93.32 (24.93)		
Medicine	188	91.64 (23.51)		
Other	84	94.36 (23.59)		
**Length of stay in China**			−0.10 (534)	0.92
Less than 1 year	393	92.75 (23.54)		
More than 1 year	143	92.97 (25.06)		

**Note**: The N did not sum up to the total sample for several variables because of missing data.

### Predictors of acculturative stress and subconstructs

Results from linear regression analysis in Table 4 shows that after controlling for covariates, country/place of origin (Africa and Asia) were positively associated with ASSIS score; being unmarried and preparedness for studying abroad were negatively associated with ASSIS scores. International students from Africa scored higher on all seven subconstructs than students from other countries. Students from Asia scored higher on rejection, value conflict, homesickness, self-confidence, and cultural competence than students from other countries. Buddhist/Hindu students scored higher than those who reported not being part of an organized religion on cultural competence, and Christian and Muslim students scored higher than others on homesickness. Unmarried students scored lower than others on value conflict and cultural competence. Lastly, preparedness was associated with all subconstructs except value conflict and homesickness.

**Table 4:** Multiple Linear Regression of ASSIS and seven components.

Variables	ASSIS	RJ	OD	IT	SC	CC	VC	HS
**Place of origin** (others [Europe/Americas/Oceania] as ref.)								
Asian countries	10.78**	**0.34** *	0.07	0.15	0.49**	0.27*	0.50**	0.30*
African countries	13.07**	**0.30** *	**0.25** *	**0.28** *	0.49**	**0.29** *	0.50**	**0.40** *
**Religion** (none as ref.)								
Buddhist/Hindu	4.75	0.11	0.14	0.09	0.09	**0.28** *	−0.04	0.21
Christian	6.74	0.19	0.25	0.18	0.11	0.08	0.11	**0.37** *
Muslim	5.78	0.07	0.25	0.10	0.02	0.16	0.07	**0.38** *
Others	2.40	−0.10	−0.15	0.15	0.18	0.29	0.19	−0.15
**Marital Status** (others as ref.)								
Unmarried	**−8.27** *	−0.24	−0.23	−0.20	−0.14	**−0.28** *	**−0.32** *	−0.24
**Preparedness** (not well prepared as ref.)								
Well prepared	**−7.58** *	**−0.28** *	**−0.25** *	−0.27**	**−0.23** *	**−0.22** *	−0.09	−0.10

Notes: One regression model for each acculturative stress measures was used. Age, gender, and length of stay were included as covariates.

*: *p*<0.05, **p<0.001.

## Discussion

In this study, we analyzed survey data from a sample of 567 international students from 94 countries who were attending universities in China. We documented the acculturative stress level among these students, proposed and empirically validated seven subconstructs of acculturative stress, and characterized the underlying 3D relationship among these constructs. Finally, we investigated how demographic and personal factors related to the overall level of acculturative stress and its subconstructs.

Stress has been recognized as a universal predictor of morbidity and mortality of many diseases [2]–[4] and as a risk factor for tobacco smoking, alcohol misuse, drug use/abuse, and other risk behaviors [2]. Along with globalization and dramatic increases in the number of international students and other cross-country mobile populations, acculturative stress has emerged as a new challenge to global health. Our findings provide new data advancing our understanding of acculturative stress and supporting therapeutic and preventive measures for stress reduction.

### Levels of and differences in acculturative stress

Findings of this study indicate that compared with international students in developed countries, the acculturative stress level among international students in China was about 10 points (*M* = 92.81[SD = 23.93] vs. *M* = 81.39 [SD = 24.66] [21] or *M* = 83.45 [SD = 25.05] [27]) higher. Students from African (*M* = 97.66, SD = 23.16) and other Asian (*M* = 92.54, SD = 22.03) countries reported more acculturative stress than students from European, American, and Oceanic countries (*M* = 81.19, SD = 27.03). The same pattern regarding region-of-origin was also reported for international students studying in developed countries in Europe, America, and other regions [21], [27]. Similar to acculturative stress observed in western countries [28], international students from African and other Asian countries in China reported higher level of acculturative stress than western countries. Since China is a typical Asian country and the cultural differences will be smaller between China and these Asian countries and larger between China and African countries. The high level of acculturative stress among the students from the other Asian countries suggest that differences in socioeconomic status rather the culture may play a larger role in acculturative stress while the increased level of acculturative stress among African students could be due to both socioeconomic status [7] and cultural factors sush as values of face and the ideology of individualism and collectivism [21]. Further research is needed to examine this issue.

### Acculturative Stress Subconstructs

Two innovative findings of this study were (1) the empirical verification of the seven subconstructs of acculturative stress: rejection, opportunity deprivation, identity threat, self-confidence, cultural competence, value conflict, and homesickness and (2) the discovery of the 3D network structure of these constructs. These findings are of great significance because they illustrate the key components of and the inner dynamics of acculturative stress. Rejection, opportunity deprivation and identity threat are three key mutually correlated components of acculturative stress. These components reflect the impact of highly correlated [17], [29] and most important external stressors [12], [22]. To treat and to prevent deleterious effects of acculturative stress on health, therapists and public health interventionists must first focus on these three stressors.

Inside the three external stressors is the second triangle of ASSIS subconstructs. These components reflect both intra-personal (self-confidence and cultural competence) and interactive (value conflict) sources of stress. Lack of self-confidence and cultural competence — and frequent culture value conflict — have been recognized as stressors [6], [15], [30]. Homesickness is the last subconstruct; it showed relatively weaker but broad connections with the other subconstructs. These findings are also informative for stress reduction. When confronting international students' stress, in addition to the three external stressors described above, therapists should discuss value conflict and attend to self-confidence and cultural competence. Homesickness is present, but comparatively the impact of this subconstruct is more independent. A correction of homesick may not lead to significant reductions in other components of acculturative stress.

### Risk and protective factors of acculturative stress

In this study we found three factors associated with higher levels of acculturative stress: lack of preparedness for studying abroad, being married, and having a religion. Preparedness has long been recognized as a protective factor of acculturative stress [19]. Its significance cannot be overemphasized in acculturative stress prevention. Interestingly, our findings suggest that being unmarried may be a protective factor for international students. Compared to international students who are married, unmarried students may have more time to focus on successfully adjusting to the new culture and environment [7]. People with a specific religion may experience difficulties in finding partners and locations for religious activities, leading to stress [24]. These factors are also to be considered in planning for stress reduction and in therapeutic services to assist students in dealing with stress.
